# A double-edged sword: iron regulation in alveolar lung epithelial repair

**DOI:** 10.1172/jci.insight.199953

**Published:** 2026-05-08

**Authors:** Ugonna Mbaekwe, Sarah Kenny, Suzanne M. Cloonan, Corrine R. Kliment

**Affiliations:** 1 Division of Pulmonary, Allergy, Critical Care Medicine, and; 2 Department of Cellular and Molecular Pathology, University of Pittsburgh School of Medicine, Pittsburgh, Pennsylvania, USA.; 3 School of Medicine, Trinity Biomedical Sciences Institute, Trinity College Dublin, Dublin, Ireland.

## Abstract

The oxygen-rich milieu of the lungs necessitates precise iron homeostasis and regulation, processes that are fundamental to pulmonary physiology but often receive limited attention. However, in recent years, dysregulation of iron homeostasis has been linked to numerous acute and chronic respiratory diseases. Here, we comprehensively evaluate the mechanisms governing iron homeostasis in the alveolar epithelium of the lung and examine how iron dysregulation contributes to impaired alveolar epithelial repair in respiratory disease. This Review focuses on the effects of iron on alveolar epithelial cell homeostasis and repair and disease pathogenesis. There will be a focus on emerging interventions designed to reestablish iron homeostasis and their potential therapeutic implications related to enhancing lung repair and limiting the progression of lung disease.

## The alveolar epithelium

The lungs maintain the largest surface area of any organ in the human body, ranging from 70 to 100 square meters (1), and are constantly exposed to external damaging stimuli, which can cause injury and subsequent disease. The lung has inherent repair processes in place to respond to damage and promote regeneration. The alveolar epithelium is a specialized layer of epithelial cells crucial for gas exchange and lung health. Alveoli are composed of a monolayer of flattened, squamous alveolar type 1 (AT1) cells that cover 95% of the surface and cuboidal alveolar type 2 (AT2) cells (2). AT2 cells produce surfactant to reduce surface tension, preventing lung collapse during exhalation. AT2 cells also serve as self-renewing progenitors that, after lung injury, differentiate into AT1 cells, which regulate gas exchange and ion transport (3).

**Figure 1:**
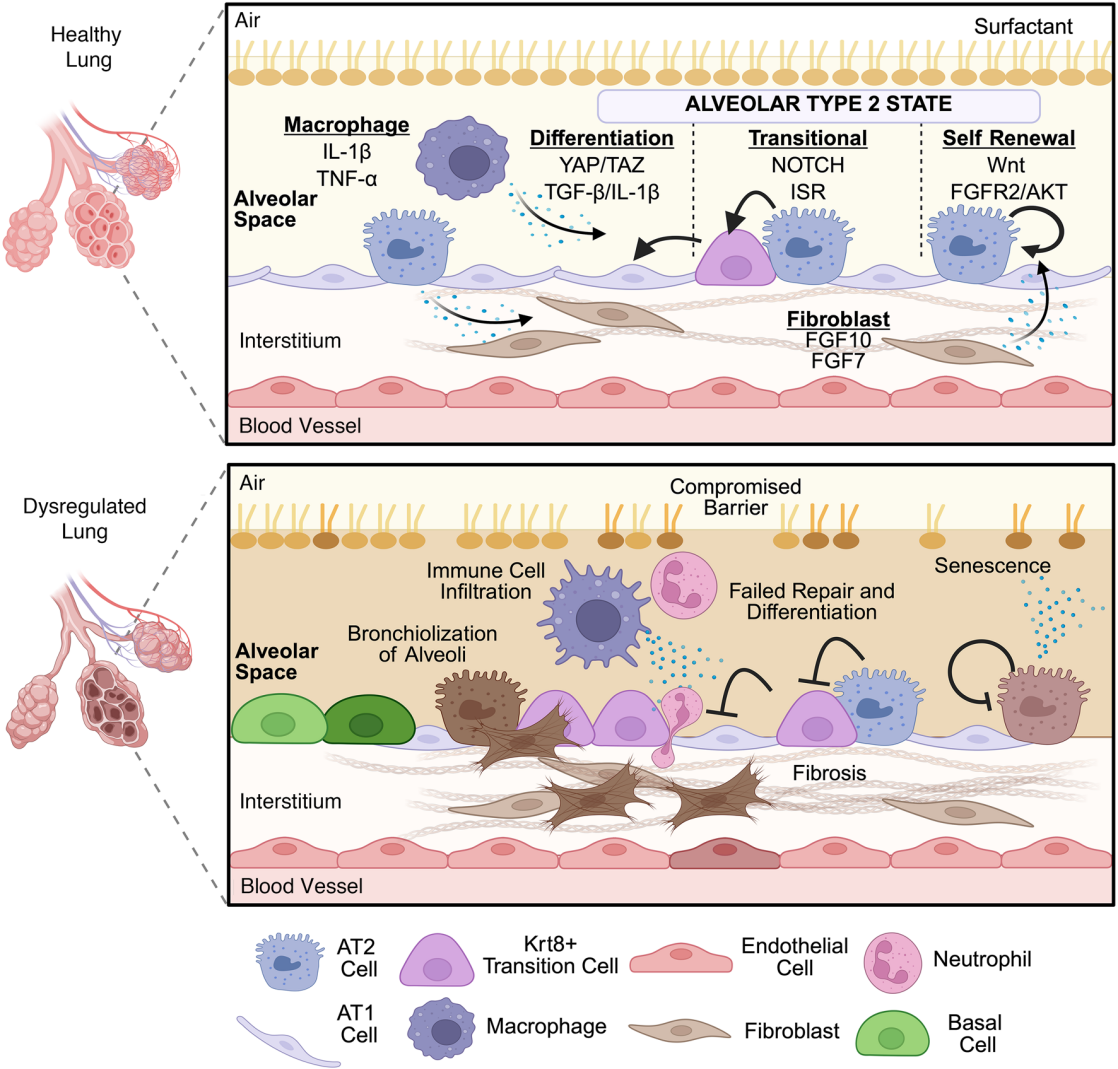
AT2 dysfunction in the lung. A schematic overview of the main interactions and signaling pathways involved in AT2 self-renewal, proliferation, and differentiation into AT1 cells. In a self-renewing state, WNT canonical and FGFR/AKT pathways primarily maintain AT2 identity and function. During differentiation, AT2 cell transition is marked by Krt8^+^ cells (also called damage-associated transient progenitor [DATP] cells). In this state, metabolic changes occur, along with integrated stress responses (ISR) and increased Notch signaling. Activation of YAP/Taz signaling promotes differentiation, and the secretion of TGF-β and IL-1β from AT2 cells in specific contexts can interact with macrophages and fibroblasts to produce factors, such as FGF7 and FGF10, that support AT2 identity. Macrophages release cytokines, such as IL-1β and TNF-α, to enhance AT2 cell proliferation and differentiation. Disruptions in the alveolar epithelial repair process can cause pathological outcomes, including bronchiolization of the alveoli, immune infiltration, increased barrier permeability, failed repair and regeneration, and the development of senescence and fibrosis within the alveolar space. Created in BioRender.

Other less abundant cell types, including mesenchymal, endothelial, and immune cells, contribute to the dynamic and spatially organized alveolar structure and defense (4–6) (Figure 1). Fibroblasts produce and remodel the ECM and alveolar architecture (6). Alveolar macrophages (AMs) serve as crucial immune cells that combat inhaled pathogens through phagocytosis and respond to environmental signals to stave off persistent inflammation and infection (7, 8). While these cell types are important in lung function, this Review will primarily focus on alveolar epithelial cells.

## Alveolar epithelium renewal and repair processes

Effective alveolar repair after lung injury is characterized by resolution of inflammation, reestablishment of the alveolar-capillary barrier, and restoration of gas exchange capability. Repair depends on timely AT2 cell proliferation for self-renewal and differentiation, which is regulated by intrinsic transcriptional factors and niche signals (2, 9–11) (Figure 1). FGFR2/AKT promotes AT2 cell survival and lineage maintenance. Loss of FGFR2 signaling in AT2 cells caused increased apoptosis, followed by compensation from remaining progenitor-like cells (12). Canonical WNT signaling maintains a WNT-responsive AT2 subpopulation (AXIN2^+^ alveolar epithelial progenitors) that preserves stemness during homeostasis and drives regenerative expansion after injury (13–15). Transcription factors, including thyroid transcription factor 1 (NKX2-1) and CEBPA, are critical for maintaining the identity, plasticity, and function of AT2 cells. NKX2-1 establishes and maintains AT2 cell fate by directly activating surfactant protein genes and repressing abnormal differentiation pathways. In addition, CEBPA is similarly enriched in mature AT2 cells, working in concert with NKX2-1 to maintain AT2 identity and to act as a gatekeeper, restricting activation of repair or transitional programs during stress (13, 16, 17).

AT2-to-AT1 differentiation is a multistep process involving both internal signaling, such as Notch signaling, and external mechanical cues that activate effectors like YAP/TAZ signaling. In AT2 cells, Notch signaling is instrumental in AT2 maintaining cell identity by promoting AT2-associated gene expression and preventing premature differentiation. At the onset of differentiation, Notch receptors undergo transcriptional suppression, thereby removing their inhibitory effect on AT1-related genes (18). During lung injury, increased tissue tension can induce dephosphorylation of YAP/TAZ, leading to nuclear translocation and subsequent activation of epithelial gene programs. These programs facilitate AT2 cell flattening and increase the expression of AT1 markers (19–21).

A complex interaction among the alveolar niche, epithelial progenitor cells, and various interstitial cells directs alveolar maintenance and repair. These supporting cells influence AT2 cell behavior through paracrine signaling and direct cell-cell interactions, modulating proliferation, differentiation, and survival during regeneration (22, 23). AT2 cells communicate with macrophages and fibroblasts through the secretion of context-dependent paracrine signals (Figure 1), such as TGF-β and IL-1β (4, 24–26). These cytokines stimulate fibroblasts to produce growth factors FGF7 and FGF10, thereby supporting AT2 cells (27–29). Additionally, secreted cytokines can induce macrophages to release IL-1β and TNF-α for further promotion of AT2 cell proliferation (30, 31). Consequently, continuous feedback loops are established, facilitating intricate multicellular interactions. Although these dynamics are important, the inherent properties of epithelial progenitor cells also significantly influence AT2 cell functioning and repair.

Alveolar repair requires substantial energy expenditure. Accordingly, AT2 cells exhibit a higher mitochondrial density and lower turnover than other lung cells, underscoring their reliance on mitochondrial function and energy metabolism (32, 33). There is growing evidence that disruptions in mitochondrial and metabolic pathways, such as calcium uptake, glutamine metabolism, glycolysis, and fatty acid oxidation, can lead to abnormal repair mechanisms and adversely affect AT2 cell reparative capacity, proliferation, and fate determination (34–38). Current evidence indicates that activation of the integrated stress response (ISR) may facilitate the transition of AT2 cells to AT1 cells (31, 38).

## Dysregulation of alveolar renewal and repair

Disruption of alveolar epithelial repair mechanisms leads to pathological outcomes, such as chronic lung disease. Homeostatic lung repair is tightly regulated through a dynamic interplay of epithelial progenitor activation, mesenchymal remodeling, immune resolution, and vascular regeneration — each precisely orchestrated to restore tissue architecture after injury. These processes can be subverted by persistent injury, aging, or abnormal signaling, leading to maladaptive repair.

Key features of dysregulated alveolar repair include loss of AT2 cell identity, impaired AT2 proliferation, and blockade of AT2-to-AT1 differentiation. Other key features include the emergence of keratin 8^+^ (*Krt8*^+^) transitional cells (also called damage-associated transient progenitor [DATP] cells) derived from AT2 cells (Figure 1). Although AT2 cells are the primary regenerative stem cells, other epithelial cells, such as club cells, contribute to alveolar regeneration (39, 40). DATP cells are generally temporary and essential for effective alveolar epithelial regeneration. DATP cells are typically recognized by increased keratin expression, a partial loss of standard AT2 markers, and partial activation of AT1 cell programs (41). Several mechanistic regulators are increasingly recognized as driving and sustaining this transitional state, including TGF-β, IL-1β, HIF1α, ISR, and senescence pathways (31, 38, 42).

However, DATP cell persistence is linked to failed regeneration and fibrosis. In mouse bleomycin (BLM) lung injury models and humans with idiopathic pulmonary fibrosis (IPF), *Krt8^+^* cells exhibit a senescence-like profile, high stress-response gene expression, and poor differentiation into AT1 cells (31, 42). AT2 cells can undergo cellular senescence, a cell cycle arrest process in response to stressors marked by increased levels of classical cell cycle arrest markers, *Cdkn1a* and *Cdkn2a* (24). Senescence further contributes to failed regeneration by promoting a senescence-associated secretory phenotype, characterized by secretion of proinflammatory factors, profibrotic mediators, and MMPs (43).

Ectopic expansion of airway-derived basal cells into the distal alveolar space indicates maladaptive alveolar repair. After severe injury — like H1N1 influenza or fibrosis — basal-like progenitors with *Tp63* and *Krt5* markers migrate into alveoli, forming abnormal structures that lack surfactant proteins and cannot support gas exchange (44). Airway secretory cells can dedifferentiate into *Tp63*^+^ basal-like progenitors, aiding alveolar regeneration during injury. However, dysregulation may cause bronchiolized, nonfunctional remodeling (45, 46). Basal cell–derived progenitors can act as emergency patches after injury, but their persistence causes maladaptive repair, displacing native alveolar progenitors and promoting pathological remodeling in chronic lung disease.

Disruption of these remodeling mechanisms, as occurs in chronic injury or aging, compromises repair fidelity, leading to abnormal lung regeneration with persistence of transitional cell states (31, 42), senescence accumulation (29, 47, 48), and failed epithelial restoration. Aberrant repair leads to pathological remodeling in chronic lung diseases like IPF and chronic obstructive pulmonary disease (COPD). Recent studies (49–54) identify iron homeostasis as a key regulator of alveolar repair, with iron imbalance driving oxidative stress, inflammation, senescence, and epithelial dysfunction that impair regeneration. This Review examines how iron influences epithelial renewal within the alveolar microenvironment.

## Iron homeostasis and regulation

Iron is an essential metal that supports multiple processes, including oxygen transport (48), DNA repair, and synthesis of heme and iron-sulfur (Fe-S) clusters, with approximately 2% of human genes encoding an iron-protein (55). However, excess free labile iron is highly toxic owing to the production of ROS (56). Thus, iron’s function is a double-edged sword, emphasizing the importance of maintaining iron balance at both systemic and cellular levels.

**Figure 2:**
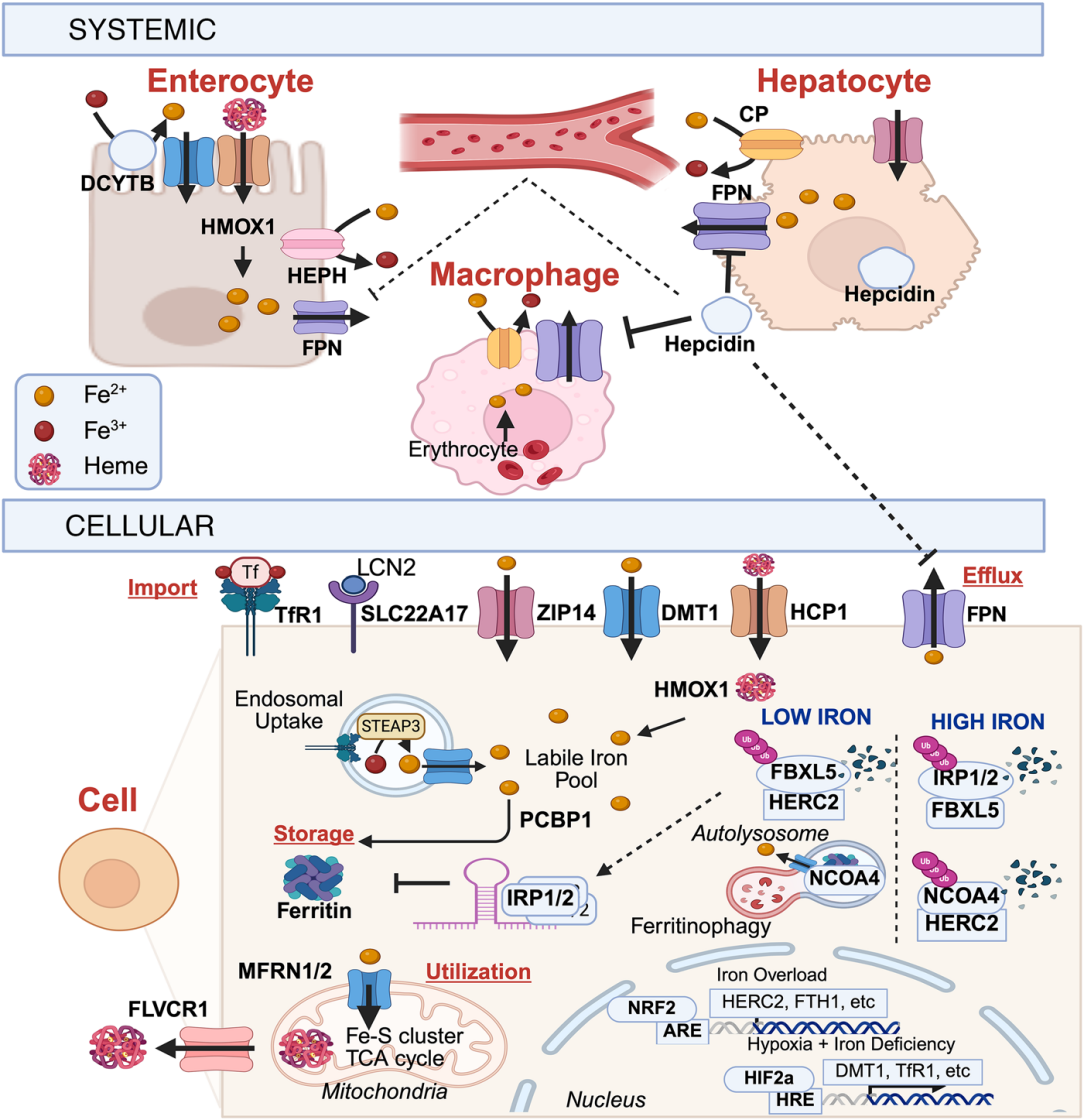
General iron homeostasis. A schematic diagram showing systemic (top) and cellular iron regulation (bottom). Systemic iron control involves various organs managing absorption, recycling, and distribution to keep levels balanced. In mammals, iron is mainly absorbed in the duodenum as both heme and nonheme iron and then transported across cell membranes and transferred in the blood to other tissues. Macrophages and hepatocytes are vital for recycling iron from red blood cells and adjusting systemic iron levels. Cellular iron regulation includes controlling iron import, export, storage, and use. Low oxygen levels and iron deficiency activate transcription factors and regulatory proteins to boost iron absorption, while iron overload triggers protective pathways to sequester, detoxify, and maintain balance. The key proteins discussed in this Review are shown at the bottom of the figure, including heme carrier protein 1 (HCP1), heme-oxygenase 1 (HMOX1), duodenal cytochrome B (DCYTB), divalent metal transporter 1 (DMT1), ferroportin (FPN), ceruloplasmin (CP), hephaestin (HEPH), transferrin receptor 1 (TfR1), lipocalin (LCN2), poly(rC)-binding protein 1 (PCBP1), mitoferrin-1 and mitoferrin-2 (MFRN1/2), leukemia virus subgroup C cellular receptor 1 (FLVCR1), hypoxia-inducible factor 2 α (HIF2α), hypoxia response element (HRE), iron-sensing E3 ligase (FBXL5), iron regulatory proteins (IRP), nuclear receptor coactivator 4 (NCOA4), and nuclear factor erythroid 2–related factor 2 (NRF2). Created in BioRender.

Systemic iron regulation involves a multiorgan network overseeing absorption, recycling, and distribution to maintain balance (Figure 2). In mammals, iron is obtained from the diet and recycled internally, mainly absorbed in the duodenum as heme and nonheme iron (57). Heme iron is transported across the plasma membrane by heme carrier protein 1 (HCP1) (58) and broken down internally by heme-oxygenase 1 (HMOX1) (59, 60). Nonheme iron is reduced from ferric (Fe^3+^) to ferrous (Fe^2+^) iron by duodenal cytochrome B and imported via divalent metal transporter 1 (DMT1) (57). Iron is exclusively exported by ferroportin (FPN), while ferroxidases such as hephaestin and ceruloplasmin oxidize iron to facilitate binding to plasma transferrin and transport to other tissues (61). Macrophages recycle iron from senescent erythrocytes in the spleen, providing a significant source of iron (62). Hepatocytes regulate iron balance via hepcidin (HAMP)/FPN, adjusting distribution as needed. HAMP, a liver hormone, binds FPN, leading to its degradation and decreasing iron release and serum iron levels (63, 64).

Cellular iron homeostasis involves the regulation of iron import, export, storage, and utilization (Figure 2). Iron import involves several different mechanisms. Transferrin-bound iron is taken up through transferrin receptor 1 (TfR1) (65), which mediates receptor-dependent endocytosis followed by iron reduction by STEAP3 and endosomal iron release through DMT1 (66). Non-transferrin-bound iron can be imported through divalent metal transporters, such as DMT1 and ZIP family members ZIP8 and ZIP14, which import iron in metabolically active and inflammatory contexts (67, 68). HCP1 internalizes heme iron, while SLC22A17 mediates the uptake of lipocalin-bound (LCN2-bound) iron during immune activation (69). Iron export is mediated by FPN, while iron storage is primarily achieved through ferritin, which buffers iron. Efficient iron delivery to ferritin is accomplished by the cytosolic iron chaperone poly(rC)-binding proteins (70). Iron utilization is primarily driven by mitochondrial processes. Mitochondrial iron import is mediated by mitoferrin-1 and mitoferrin-2 (MFRN1/2) (49, 71, 72), which deliver iron for heme biosynthesis, Fe-S cluster assembly, and metabolic enzyme function. Excess heme can be exported from the cell via feline leukemia virus subgroup C cellular receptor 1 (FLVCR1) (73, 74) to maintain heme-iron balance.

Cellular iron levels are regulated at the level of gene expression. During hypoxia or iron deficiency, stabilization of HIF2α (75) activates transcription of hypoxia response element–containing genes that enhance iron uptake and utilization. Ubiquitin ligase HERC2 posttranscriptionally degrades iron-sensing ligase (FBXL5), leading to stabilization of iron regulatory proteins (IRPs) (76). In low iron states, IRP1 lacks its Fe-S cluster and switches to its iron response element–binding (IRE-binding) form (77). IRP1 stabilizes iron import protein-encoding mRNAs and represses translation of ferritin to reduce iron storage (78). IRP2, normally degraded by FBXL5, stabilizes when FBXL5 is degraded, increasing iron uptake and reducing storage (79, 80). Nuclear receptor coactivator 4–mediated (NCOA4-mediated) ferritinophagy is enhanced to liberate stored iron through ferritin degradation in lysosomes (81). Conversely, in conditions of iron overload, activation of the nuclear factor erythroid 2–related factor 2 antioxidant pathway induces the expression of genes involved in iron sequestration, detoxification, and redox defense., FBXL5 is posttranscriptionally stabilized and promotes degradation of IRPs. When bound to a Fe-S cluster, IRP1 converts into a cytosolic aconitase and loses the ability to bind IREs. IRP2 is degraded by FBXL5, while HERC2 degrades NCOA4 to limit ferritinophagy (82).

**Figure 3:**
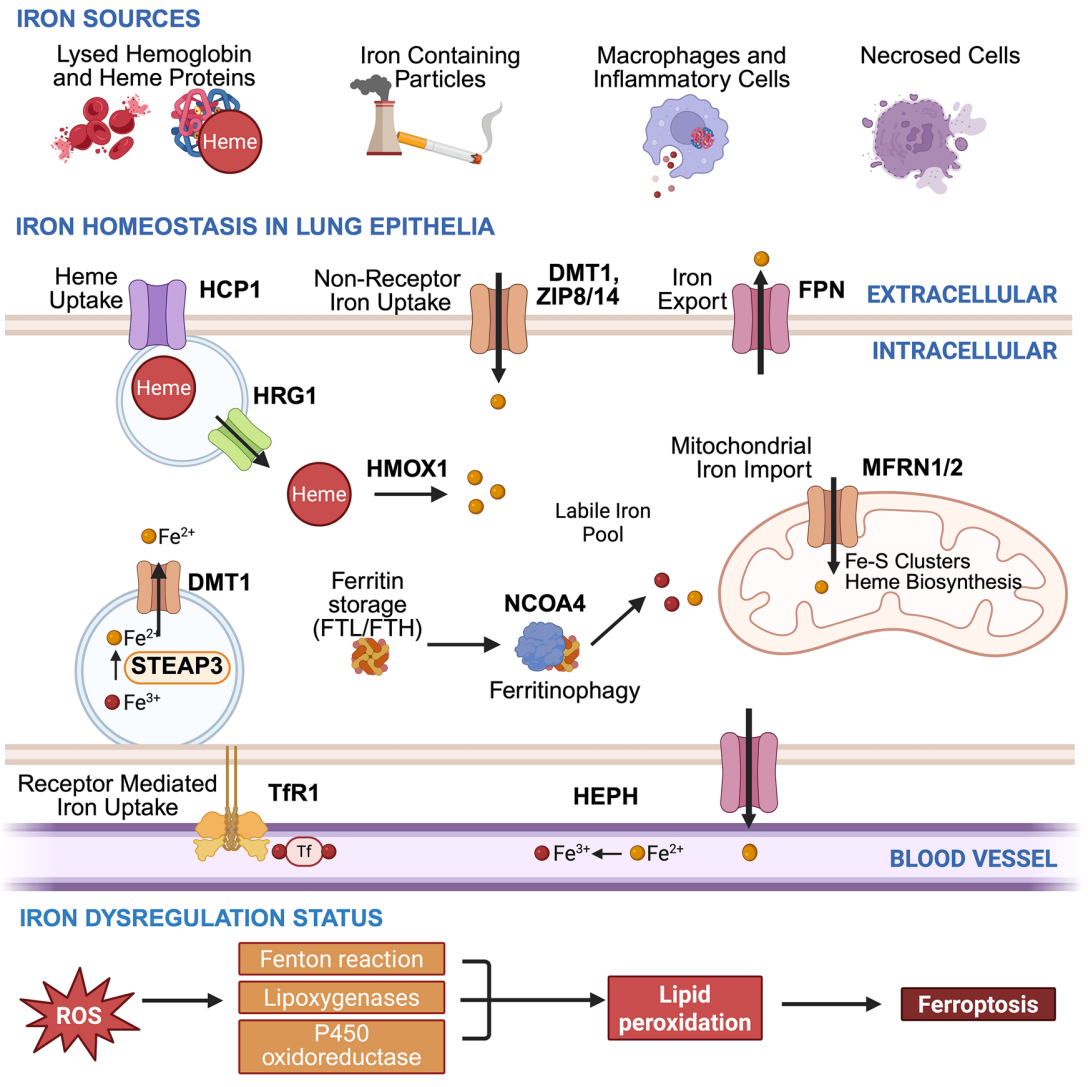
Lung iron homeostasis. A schematic illustrating lung iron sources and regulation (top) and how iron dysregulation triggers ferroptosis (bottom) in lung epithelial cells affected by disease. The lung depends on endogenous sources, like cellular recycling, and exogenous sources, such as particulate matter, which increases oxidative stress and inflammation. Excess iron results in ROS, lipid peroxidation, and ferroptosis when antioxidants are overwhelmed. Created in BioRender.

Tight regulation of cellular iron is important for meeting metabolic needs and controlling redox balance (Figure 3). Unregulated Fe^2+^ reacts with ROS through a Fenton reaction to generate highly reactive hydroxyl radicals (83–85) and catalyzes the oxidation of polyunsaturated fatty acids in membranes to form lipid hydroperoxides (86). This process sensitizes cells to an iron-dependent form of cell death called ferroptosis. Ferroptosis is driven by the lethal accumulation of lipid peroxides (87). When antioxidant defenses, especially glutathione peroxidase 4 (GPX4), are overwhelmed or impaired, lipid peroxides accumulate unchecked, triggering cell death. Ferroptosis is particularly relevant in tissues with high oxidative stress and iron turnover, like the lung, where dysregulated redox balance and iron overload contribute to both acute and chronic injury (88).

## Alteration in iron homeostasis and regulation in the lung

Although not traditionally viewed as an iron-handling organ, cells in the lung require iron. The lung is continuously exposed to endogenous and exogenous iron (89). Cellular iron mainly comes from endogenous sources like transferrin-bound iron, macrophage recycling, and heme-iron from alveolar hemorrhage (Figure 3). Exogenous iron sources include cigarette smoke (CS), which at 0.06% iron, contributes minimally, and PM2.5, which contains 0.5%–5% to 20%–43% iron, with high levels near industrial sites (90–92). Increases in lung iron from exogenous sources are primarily due to particle-bound iron complexes that promote inflammation, oxidative stress, and macrophage iron retention.

Although systemic HAMP regulates overall iron balance, local lung mechanisms in epithelial cells and AMs fine-tune iron handling and influence disease progression. Dysregulation can disrupt redox balance and hinder lung repair. Iron buildup in the alveolar space, particularly as heme-derived compounds, can compromise barrier integrity and elevate epithelial permeability, underscoring the importance of strict iron regulation in the lung (93, 94). Heme-bound iron induces oxidative stress and creates a prooxidant pool in epithelial lining fluid (ELF) that is exacerbated by CS, hemolysis, or lung damage. In contrast, transferrin and ceruloplasmin in ELF act as antioxidants by binding nonheme iron but do not neutralize heme species–like free heme and hemoglobin (95).

Iron metabolism in the alveolar space plays an additional role in host defense, as AMs and epithelial cells regulate iron supply in response to pathogens. Recently, in vitro studies show that AT2-like cells adjust their iron metabolism based on intracellular versus extracellular pathogens, suggesting a role for AT2 cells in regulating lung-iron during infection (96). AMs modify iron handling through iron sequestration as part of nutritional immunity (97, 98). Dysregulated iron in AMs is associated with impaired bacterial clearance and increased exacerbations of chronic lung disease (97, 98).

AMs are well documented for their role in iron regulation in pathological contexts (99, 100). AMs are involved in iron trafficking, use iron for metabolism in phagocytosis, serve as iron reservoirs, and can be independent of the HAMP/FPN axis (101, 102). In response to LPS, AMs upregulate endogenous HAMP expression, highlighting their ability to influence iron metabolism during inflammation (102). Disruption of the local HAMP/FPN axis causes iron deficiency in AMs, impairing phagocytosis. Thus, intracellular iron serves as a metabolic fuel for efficient phagocytosis in AMs. Notably, in IPF, TfR1-deficient AMs were more abundant and associated with decreased survival (103). Dysregulation of iron metabolism in AMs creates a local iron-rich and oxidative microenvironment. This environment directly affects lung epithelial cells by increasing extracellular iron in the alveolar space that is available for import, altering epithelial iron export activity through HAMP, and triggering inflammatory signaling (99). Paracrine signaling, such as SPP1, promotes cross-talk between AT2 cells and macrophages, driving the recruitment of immune cells and maladaptive cell differentiation (50).

There is less documentation of iron regulation in lung epithelial cells in pathological contexts. Systemic iron dysregulation partly contributes to cellular iron regulation in the lung (51, 104). Notably, point mutations in FPN result in iron accumulation in the alveolar region and thickening of the air-blood barrier with AT2 cell hypertrophy and hyperplasia (51). Global HAMP deficiency alters baseline iron in the lung but does not affect smoke-induced injury (105). HAMP/FPN dysfunction affects the alveolar airspace, but lung epithelial cells can facilitate iron uptake pathways, thereby reducing redox-mediated damage (95, 106). Cellular iron uptake receptors, including TfR1, ZIP8/14, and DMT1, are expressed in the lung epithelium (99, 107, 108). While these importers are found in lung epithelial cells, there is no conclusive evidence to confirm their specific role in maintaining steady-state iron regulation. The mechanisms underlying alternate iron uptake pathways in lung epithelial cells, such as TfR2 and LCN2, remain uncertain. In Tfr2-mutant mice, systemic iron overload leads to significant iron accumulation in the lungs, particularly in the airway basement membrane and tissue macrophages (109), suggesting a role in lung epithelial cells. In COPD, the presence of LCN2 and ferritin in bronchoalveolar fluid (BALF) suggests potential mechanisms for iron absorption (110), with AT2 cells reported to internalize native ferritin (111). Ferritin light chain (*FTL*) is one of the most abundant transcripts in lung tissue, with both ferritin subunits localizing to alveolar cells (112, 113). However, it remains unclear whether AT2 cells take up iron-bound ferritin and if other lung cell types use similar endocytic mechanisms, as excess iron in lung cells is either stored in ferritin or exported by FPN (114).

Despite limited research, given their unique position at the air-blood barrier, it is likely that AT2 and AT1 cells use multiple iron uptake and transport pathways to regulate iron levels in the alveolar space. Direct regulation of iron in AT1 cells remains underexplored compared with that in AT2 cells. However, AT1 cells are known to be prone to ferroptosis due to oxidative stress. Excess heme-derived iron was shown to be a potent mitochondrial toxin in AT1 cells, impairing mitochondrial metabolism and membrane integrity (94). Moreover, recent work highlights a possible affinity for iron regulatory genes in alveolar repair and alveolar cell identity (115, 116) as well as the association between iron buildup in the alveolar space and restrictive lung disease (104). The alveolar epithelium is vulnerable to such disruptions in iron levels in the alveolar space. Elevated iron levels are associated with alterations in surfactant biophysical properties (117, 118), mitochondrial iron dysregulation in AT2 cells promotes oxidative stress, and age-related changes in iron regulatory gene expression may impair their regenerative capacity (49, 116). Together, these studies further emphasize the important role of iron handling in maintaining alveolar function.

## Iron as a determinant of alveolar epithelial fate and repair

**Figure 4:**
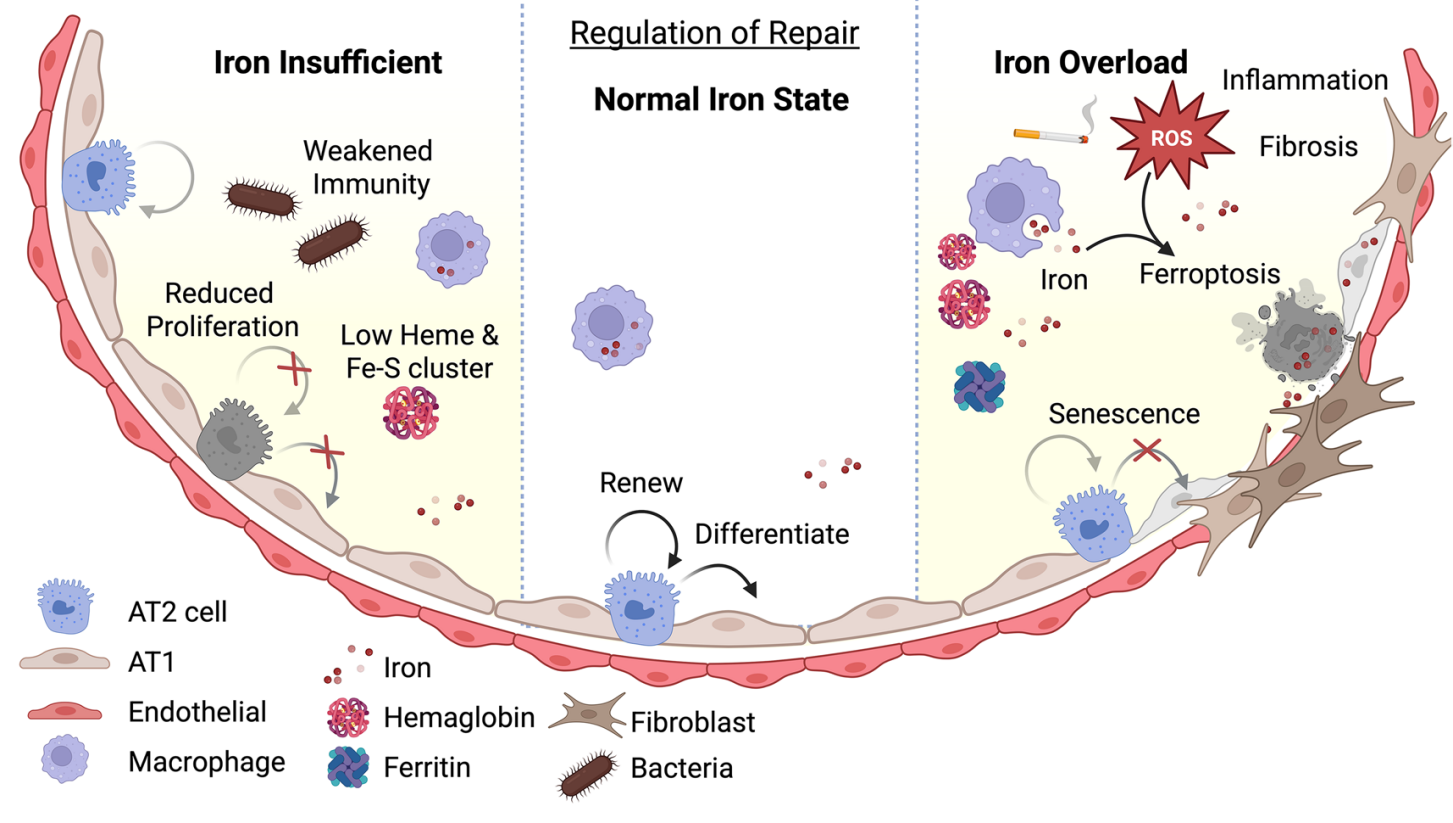
Role of iron regulation in alveolar repair. A schematic illustrating how iron levels influence cell fate and lung repair in the alveoli. In iron deficiency, alveolar epithelial cells experience metabolic stress, resulting in mitochondrial dysfunction and decreased surfactant production. This stress reduces their capacity to self-renew and differentiate. In alveolar macrophages, stress triggers premature senescence and impairs iron recycling, which weakens phagocytosis and immune responses, leading to immune exhaustion during chronic iron deficiency. Conversely, under normal iron levels, alveolar cells manage iron flux, reducing senescence and ferroptosis, while maintaining immune function. Iron overload causes oxidative stress, mitochondrial dysfunction, ferroptosis, epithelial loss, and tissue damage, impairing regeneration and differentiation. Excess iron in macrophages hampers antimicrobial activity, promotes inflammation and fibrosis, and raises the risk of chronic lung disease. Created in BioRender.

Numerous lung cells contribute to the development of lung disease; still, there is increasing evidence that iron homeostasis influences AT2 renewal, differentiation, and repair capacity through mitochondrial dysfunction, ferroptosis, and senescence (Figure 4). Given that AT2s are progenitor cells, evidence from broader themes of stem cell research can be informative. A study from Kao and colleagues shows that labile iron levels serve as a rheostat for cellular quiescence in mitotic hematopoietic stem cells (HSCs), suggesting that iron is crucial for determining stem cell fate (119). Variations in cytoplasmic iron levels influence the equilibrium between self-renewal and differentiation in adult stem cells, determining HSC state. Low intracellular iron favors quiescence and self-renewal, preserving long-term regenerative capacity, while high iron triggers metabolic and oxidative stress pathways, pushing HSCs toward differentiation or exhaustion (119).

A 2025 study by Zhuang et al. shows that iron homeostasis regulates AT2 cell renewal, differentiation, and lung repair in lung adenocarcinoma cancer models. Aging-related functional iron deficiency in AT2 cells reduces self-renewal and regenerative abilities (52). Although the precise intracellular iron pools and trafficking routes in isolated AT2 cells have not been fully assessed, the study shows that aging rewires iron handling in AT2 cells by inducing nuclear protein 1 (NUPR1), which upregulates LCN2, lowering bioavailable iron, limiting stemness, and inhibiting tumorigenesis. Knocking out the NUPR1/LCN2 axis or iron supplementation in aged AT2 cells can enhance renewal and rescue stemness but also promote tumorigenesis in aged lungs (52). Collectively, these studies emphasize the importance of maintaining an appropriate iron balance for effective AT2 cell–mediated lung repair. Disruptions in iron homeostasis directly impair epithelial cell regeneration, ultimately hindering alveolar repair in chronic lung diseases.

## Iron dysregulation in chronic lung disease

Iron dysregulation is linked to chronic lung disorders, including IPF and COPD, in which excess iron leads to oxidative stress, ongoing epithelial damage, impaired regeneration, and accelerated disease progression. Exposure to exogenous iron increases susceptibility to respiratory diseases, as seen in individuals working in iron ore mines, who often develop persistent respiratory symptoms even after exposure ends (120).

While iron dysregulation affects lung epithelial repair and lung disease progression in several lung diseases (Table 1), this Review focuses specifically on COPD and IPF. Even though COPD and IPF have distinct pathologies, the role of iron homeostasis in epithelial lung repair is well documented, with evidence of sustained imbalances in iron metabolism. These imbalances compromise AT2 regenerative capacity, promote oxidative stress, induce epithelial ferroptosis (121, 122), and disrupt epithelial barrier integrity, ultimately impairing effective alveolar regeneration and perpetuating disease.

### IPF.

IPF is a chronic, progressive interstitial lung disease of unknown etiology, characterized by irreversible fibrosis and a rapid decline in lung function (123, 124). Iron accumulation has been observed in lung tissue and BALF of individuals with IPF, with increased iron levels negatively correlating with disease severity (109). Additionally, mitochondrial ROS levels in whole lung tissue from individuals with IPF are substantially increased (125). Iron overload is increasingly linked to the development of fibrosis in the lung and other organs, such as the kidney (109, 126). Genetically, a higher frequency of polymorphisms in the HFE gene, which underlies hereditary hemochromatosis, has been observed in individuals with IPF and is associated with increased iron-dependent ROS generation in BALF-derived cells (127).

BLM administration is a standard experimental model of pulmonary fibrosis; however, the role of iron in BLM-induced injury is complex and context dependent. BLM itself functions as an iron chelator and requires iron to generate ROS and induce DNA damage (128). This property of BLM does not preclude the possibility that exogenous or dysregulated iron levels can modulate the severity of lung injury. In support of this notion, Maus et al. demonstrated that intratracheal administration of iron alone can induce acute lung injury and fibrosis in mice (126). Unfortunately, the literature on the effects of iron in BLM-induced injury is not entirely consistent. For example, systemic iron overload, via a high-iron diet, in HAMP-knockout mice did not exacerbate BLM-induced fibrosis (129), but fibrotic responses were reduced in iron-deficient hamsters subjected to BLM (130). Tfr2-mutant mice, which exhibit systemic iron overload, have markedly worsened fibrosis following intratracheal BLM exposure (109). Treatment with iron chelators at the time of BLM injury onset mitigated lung fibrosis, further implicating iron as a pathogenic factor in this model (53). These findings suggest that the source, compartmentalization, and timing of iron availability — rather than total body iron status — may critically influence disease outcomes. This variability underscores the importance of the experimental context when interpreting iron’s contribution to fibrosis.

As noted above, AMs are the predominant iron^+^ cells in IPF (131, 132). In the BLM-induced pulmonary fibrosis mouse model, intracellular iron levels are increased not only in AMs, but in AT2 cells and fibroblasts (49, 53, 133, 134). Studies of the alveolar epithelium suggest that cellular and mitochondrial iron accumulation in AT2 cells promote ferroptosis (53) and mitochondrial dysfunction (49), respectively. However, AT2 cell iron status was evaluated in immortal murine AT2-like MLE-12 cells or whole lung tissue, limiting specific conclusions on AT2 cell iron status. Nonetheless, given the high lipid content in AT2 cells and their role in surfactant production, it is plausible that disruptions in AT2 cell iron could heighten lipid peroxidation. Notably, Pei et al. demonstrated that iron chelation and inhibition of ferroptosis with liproxstatin inhibited the development of BLM-induced fibrosis in a mouse model (135). However, the use of intraperitoneal liproxstatin administration does not allow definitive conclusions about the local cell types targeted by the treatment.

Despite emerging research on the role of iron in fibrotic lung remodeling, current knowledge centers on fibroblasts and immune cells (134), with a relatively limited understanding of how iron dysregulation directly affects alveolar epithelial cells, particularly AT2 cells. This represents a critical gap, as AT2 cell dysfunction and failed repair are both hallmarks of IPF. Additional studies are needed to understand how iron-induced cell fates, such as ferroptosis and impaired iron trafficking, compromise alveolar epithelial repair and reveal potential therapeutic targets.

### COPD.

COPD is characterized by chronic inflammation, airway epithelial remodeling, destruction of alveolar epithelium, and dysfunctional repair processes. The most prominent risk factors for COPD include CS or other inhaled environmental exposures, age, and genetic factors. GWAS have identified significant SNPs in *IREB2* (which encodes IRP2), in severe COPD cases, suggesting *IREB2* as a COPD susceptibility gene (136). IRP2 levels are increased in lung tissues from patients with COPD (136). In lung epithelial cells, upregulation of *IREB2* via circular RNA circSAV1 increased cellular iron and subsequent ferroptosis (137). IRP2-deficient mice are protected against CS exposure–induced experimental COPD as a result of limited mitochondrial iron loading (54) and from developing pulmonary inflammation in a tracheal banding surgery airway obstruction model (138). Porphyria, a genetic disease of dysregulated iron metabolism, is associated with an increased risk of COPD (139).

Systemic iron dysregulation is present in COPD, with elevated intracellular iron deposits detected in lung tissues from patients with very severe COPD (GOLD 4 stage) compared with healthy individuals acting as controls (140). Both current and former smokers exhibit elevated iron and ferritin levels in BALF (90) and AMs (141–144) compared with nonsmoking individuals acting as controls. COPD exhibited an increase in the quantity of iron and percentage of iron^+^ AMs, and this increase correlated with disease severity and airflow obstruction (144). Within the COPD cohort of the Subpopulations and Intermediate Outcome Measures in COPD Study (SPIROMICS), iron and ferritin were elevated in the BALF of both patients with COPD and smokers without COPD (110). This increase in iron markers in BALF correlated with disease exacerbation risk; however, the same correlation was not observed with plasma markers. Serum levels of HAMP were increased in individuals with mild-to-moderate COPD and those with COPD exacerbations (145, 146); however, HAMP was decreased in severe (GOLD 3/4) COPD (147).

While iron overload appears to be present at the cellular and tissue level in COPD, systemic iron overload is more variable. Several studies have demonstrated higher serum iron, ferritin and LCN2 in individuals with COPD compared with individuals acting as controls (91,146, 148), while other reports have not observed similar changes (149) or demonstrated systemic anemia (89). Studies suggest that systemic iron deficiency is present in a proportion of patients with COPD (10%–40%) (145, 148, 150, 151), despite possible iron overload at the lung tissue or cellular level.

In CS-exposed mice and other experimental COPD models, iron is increased in lung tissue and BALF (81, 138). In mice, exposure to environmental iron powder intensifies lung inflammation, oxidative stress, and remodeling, which are more pronounced when underlying lung damage is present (152). Given the presence of cellular iron overload, ferroptosis has been implicated in COPD pathogenesis, as evidenced in human COPD lung tissues (153) and AT2 cells (154). Additionally, CS exposure in both in vivo and in vitro models induces labile iron accumulation and ferroptotic cell death (122, 155). Furthermore, CS-induced ferroptosis was inhibited both by iron chelators, such as deferoxamine (DFO), and ferroptosis inhibitors, ferrostatin-1 and liproxstatin-1 (122).

Comparisons between COPD and IPF highlight both shared and distinct mechanisms of alveolar injury and repair. In both diseases, iron loading occurs in lung tissue and BALF, with strong evidence of localization to AMs. While ferroptosis signatures have been suggested in AT2 cells, direct evidence in primary cells in either condition is lacking. Both diseases feature transitional epithelial states, with AT2 cells consistently described as senescent, suggesting that iron accumulation and senescence may interact to impair regenerative and repair capacity. While iron regulation shows therapeutic promise, its direct effect on AT2 function remains poorly defined. Clarifying how iron overload alters AT2 behavior over time may uncover intervention points to prevent irreversible lung damage.

## Therapeutic opportunities targeting iron pathways for lung regeneration

As highlighted in this Review, iron deposition appears to be a key pathological feature of IPF and COPD, with environmental exposures, such as CS, linked to elevated lung iron levels. Genetic factors may further influence iron handling and shift disease susceptibility. Thus, current data support the exploration of therapies that target iron homeostasis.

Iron chelators are compounds (natural or synthetic) that bind iron to reduce iron availability and limit related complications. In the United States, 3 iron chelators are FDA approved: DFO, deferiprone (DFX), and deferasirox (156). These drugs have historically been used to treat iron overload in hepatic, hematologic, and cardiac disease in the absence of anemia (157, 158). DFO is an intravenous agent that chelates extracellular iron and binds to ferritin. While not commonly used to treat COPD, DFO is the standard treatment for acute iron overload (159). DFO has been shown to improve CS-induced inflammation, lung injury, and ferroptosis in lung epithelial cells (54) and reduce downstream ferroptosis in nasal and lung epithelium in CS-induced injury models (122, 160).

In contrast, DFX is orally available and chelates intracellular iron. In a COPD mouse model induced by CS, DFX reduced lung inflammation and injury (54) and reduced iron load and CS-induced ferroptosis in lung epithelial cells (122). These benefits could be achieved through inhaled delivery, providing direct exposure to the lungs. While iron chelation and ferroptosis inhibition have demonstrated benefits in animal models of chronic lung disease, it remains unclear how different cell types contribute to this protection, how lung repair is affected, and how efficient these drugs may be in human disease. Furthermore, there could be different responses in early versus late disease and active versus former smokers with established disease.

The therapeutic efficacy of iron chelators is partly hindered by their short half-life and cytotoxicity. In the cancer field, the use of nanomaterials to prolong half-life, enhance cellular delivery, and increase chelation capacity has been proposed (161, 162). For some iron-related pathways, such as those involving high versus low HAMP in COPD, further studies are needed before targeting them with therapeutic interventions. Additional therapies could target specific components of iron metabolism beyond systemic chelation, such as mitochondrial pathways involved in Fe-S cluster assembly or heme synthesis. Mitochondria-targeted iron chelators are being developed (163), raising important considerations, such as differential effects of modulating cytosolic versus mitochondrial iron pools.

While iron chelation has been proposed as a therapeutic strategy to counter iron overload in the lung, its clinical utility remains complex and highly context dependent. Treatments with iron chelators present particular challenges, as differences in dose, route, and duration of administration can lead to inconsistent or even contradictory outcomes of increased infection risk, exacerbated anemia (89, 164), and impairment of essential cellular processes that rely on iron, including mitochondrial respiration, DNA synthesis, and cellular proliferation. There are studies showing that iron chelators induce mitophagy (165, 166). Iron is central to AT2 cell function and by extension surfactant production, given that surfactant synthesis and secretion rely on AT2-specific lamellar body dynamics and are powered by mitochondrial energy metabolism. Disruption of lipid metabolism in AT2 cells via CS (167) reduces key surfactant phospholipids and increases markers of ferroptosis, highlighting a potential link between iron imbalance, oxidative damage, and impaired AT2 function.

Therefore, approaches that more selectively modulate downstream consequences of iron dysregulation are gaining attention. Interventions targeting cell death pathways downstream of iron, such as ferroptosis, hold promise for these chronic lung diseases (88, 122). Targeting ferroptosis not only addresses iron dysregulation, but may preserve AT2 cell viability and progenitor capacity. Pharmacological blockade of ferroptotic pathways — through lipid peroxidation inhibitors, iron regulators, or GPX4 stabilizers — has the potential to protect AT2 cells from oxidative injury, preserve their self-renewal and differentiation capacity, and support epithelial regeneration. Moving beyond systemic approaches, more targeted strategies — such as localized delivery or combination therapies — may improve efficacy and reduce off-target effects. In this context, emerging approaches such as iron-activated senolytic prodrugs, which selectively exploit elevated iron levels in senescent cells, offer promising avenues for therapy (168). Combining inhaled iron chelators with senolytic agents could represent a future, more precise therapeutic strategy to modulate iron homeostasis and improve outcomes in lung disease.

## Future directions and gaps

Although iron regulation is critical in chronic lung diseases such as IPF and COPD, many questions still remain. What causes pulmonary iron overload — whether it results from impaired export, excessive uptake, HAMP/FPN dysregulation, or vascular leakage — and how do these mechanisms differ by disease type or stage? Is iron dysregulation a cause or a consequence of the disease? It is unknown whether iron overload accelerates disease or worsens existing conditions, as excessive iron is also naturally linked to aging (169, 170). Additional studies are needed to investigate the roles of iron in both acute and chronic conditions. Diseases like IPF and COPD likely involve ongoing iron accumulation in AT2 cells, whereas acute injuries involve rapid increases in iron and heme due to barrier breakdown, which trigger protective responses, including upregulation of ferritin and HMOX-1. A major limitation in current research is the direct isolation of AT2 cells to investigate their iron metabolism. Although Maus et al. suggested that iron accumulates within AT2 cells, they did not identify the classical pathways typically associated with iron overload (126). As a result, it is still unclear how iron affects the function of healthy AT2 cells, transitional states, or AT1 cells. Existing signatures may be confounded by the heterogeneity of epithelial cell populations rather than reflecting true AT2-specific biology.

Knowledge gaps extend beyond the alveolar epithelium; other lung regions with progenitors, such as basal, club, and bronchioalveolar stem cells, may be affected by iron dysregulation. Advanced techniques like single-cell transcriptomics can help identify vulnerable cell populations. Understanding these time- and cell-specific changes is key to developing targeted therapies, as strategies effective in acute settings could be harmful in chronic diseases. Filling these gaps is essential for creating precise treatments that enhance iron regulation, support alveolar repair, and slow lung disease progression.

## Conflict of interest

SMC is supported by a Research Ireland Future Research Leaders (FRL4862) award and a Research Ireland Laureate Award (IRCLA/2022/3619). SMC holds a patent (US 10,905,682 B2) for the use of mitochondrial iron chelators for the treatment of COPD.

## Funding support

This work is the result of NIH funding, in whole or in part, and is subject to the NIH Public Access Policy. Through acceptance of this federal funding, the NIH has been given a right to make the work publicly available in PubMed Central.

- NIH National Heart, Lung, and Blood Institute (R01HL168050) to CRK.
- Burroughs Wellcome Fund Career Award for Medical Scientists to CRK.
- Research Ireland Future Research Leaders (FRL4862) award to SMC.
- Research Ireland Laureate Award (IRCLA/2022/3619) to SMC.

## Figures and Tables

**Table 1:**
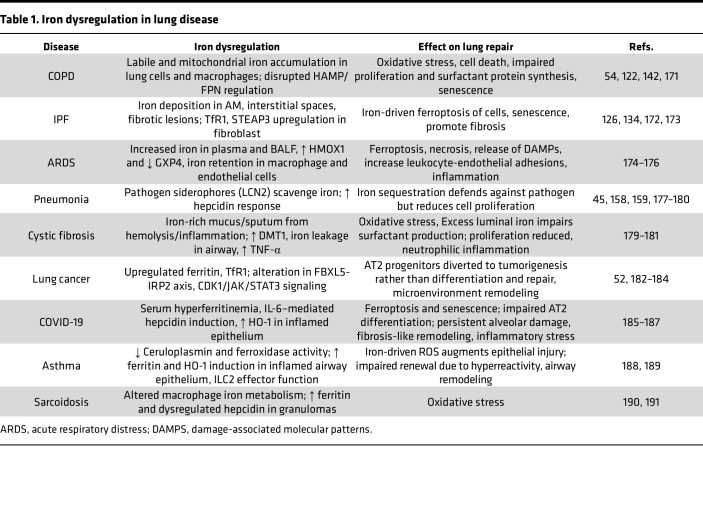
Iron dysregulation in lung disease

## Version 1 05/08/2026

Electronic publication
